# An Expert Opinion on “Glycemic Happiness”: Delineating the Concept and Determinant Factors for Persons with Type 2 Diabetes Mellitus

**DOI:** 10.3390/clinpract11030071

**Published:** 2021-08-20

**Authors:** Sanjay Kalra, Ashok Kumar Das, Gagan Priya, Ameya Joshi, Hitesh Punyani, Nareen Krishna, Kumar Gaurav

**Affiliations:** 1 Department of Endocrinology, Bharti Hospital and BRIDE, Karnal 132001, India; brideknl@gmail.com; 2 Department of Endocrinology and Medicine, Pondicherry Institute of Medical Sciences, Puducherry 605014, India; ashokdas82@gmail.com; 3 Department of Endocrinology, Fortis Hospital, Chandigarh 160011, India; gpriya77@gmail.com; 4 Department of Endocrinology, Bhaktivedanta Hospital, Mumbai 401107, India; ameyaable@gmail.com; 5 Department of Medicine, Chaitanya Cardio Diabetes Centre, New Delhi 110026, India; hiteshpunyani@gmail.com; 6 Department of Medical Affairs, Dr. Reddy’s Laboratories Limited, Hyderabad 500034, India; kumargaurav2@drreddys.com

**Keywords:** diabetes, psychological impact, glycemic happiness, glycemic happiness scale, quality of life, psychosocial impact

## Abstract

The importance of the psychological impact of diabetes is globally well-documented. Evidence suggests that there is a high level of psychosocial burden of diabetes in India. Moreover, there is a lack of relevant knowledge among the patients and caregivers regarding the psychological impact of diabetes and how to cope with it, as compared to the majority of other countries. “Happiness of the patient” is an essential component of diabetes management, which potentially affects the treatment outcome, treatment adherence, self-care, and lifelong management of diabetes. Although several validated tools and scales exist for measuring psychological outcomes both in patients and physicians, tools to assess “happiness in diabetes care” are still lacking. With this background, an expert group meeting was held in India in September 2019, involving nine expert diabetologists and endocrinologists across the country to discuss the concept of “glycemic happiness”. This article summarizes the expert opinion on the factors affecting psychological outcomes in diabetes, introduces the concept of glycemic happiness, describes available scales and tools to measure general happiness, and delineates the five sets of questionnaires developed with questions that may help correlate with “glycemic happiness”. The questionnaires are based on a five-point Likert method. The experts also discussed and decided upon the study design for a proposed observational survey to assess glycemic happiness of persons with type 2 diabetes mellitus (T2DM) based on the developed five sets of questionnaires. Given the huge burden of diabetes in India, the introduction of the concept of glycemic happiness will help in the optimization of diabetes care in the country.

## 1. Introduction

Diabetes is a chronic metabolic disease, which not only affects physical health but also impacts the social and mental well-being of people living with it. The worldwide prevalence of diabetes has been increasing consistently, reaching endemic proportions over the last few decades [1]. In 2019, approximately 463 million adults were living with diabetes. By 2045, this number is projected to reach 700 million [2]. According to recent data, the prevalence of diabetes in the adult population of India is 8.9%, with 77 million cases [3]. India is sitting on a diabetic volcano, with the number of persons with diabetes estimated for 2030 predicted to be attained by 2020 [1].

Evidence indicates that diabetes and its complications are strongly associated with emotional and psychological problems, including depression, emotional stress, poor eating habits, poor adherence, and fear of hypoglycemia. Addressing the psychological needs in such individuals leads to improved diabetes outcomes and results in lowered comorbid psychological disorders [1]. A common burden associated with diabetes is diabetes distress, which stems from the perceived inability in coping with the various demands and challenges of living with diabetes. Diabetes distress can be diagnosed using a validated tool, such as the diabetes distress scale (DDS) [4]. One of the causes of diabetes distress is insulin distress, which is defined as the emotional response of a person who has been advised to use insulin. Insulin distress is characterized by dejection or denial, discomfort, and apprehension due to a perceived inability to cope with insulin therapy [5]. Strengthening the coping skills of persons with diabetes helps in the management of diabetes distress and insulin distress, which, in turn, contributes to the emotional and physical wellbeing of the person [4,6]. In other words, achieving optimum satisfaction levels in terms of diabetes control among patients is essential for improving patient-centric outcomes in diabetes care.

Health is a construct of biological, mental, and emotional well-being. The biopsychosocial model of healthcare is being increasingly incorporated into modern medicine. Diabetes care should not focus just on amelioration of physical symptoms and reducing the risk of complications, but also focus on the promotion of well-being [7]. Healthcare providers (HCPs) commonly approach diabetes management only in terms of achieving or implementing physical or biomedical targets. This is one of the major challenges in optimal diabetes care. The World Health Organization (WHO) emphasizes the importance of bringing emotional and mental aspects of health into consideration, in addition to physical health. Many persons living with diabetes tend to be unhappy with the care and services that they receive from their HCPs and caregivers, which can affect their adherence to appropriate self-care behavior. On the contrary, the HCPs are often dissatisfied with the lack of appropriate self-care undertaken by persons with diabetes. Therefore, the routine practices of caregivers/healthcare providers/nurses/diabetes counselors to ensure glycemic happiness among patients will cumulatively enhance patient satisfaction, which is essential for improving diabetes outcomes. Being happy, or euthymia, should, therefore, be considered as a target in diabetes care [8]. However, there is no appropriate definition of “glycemic happiness” in persons living with diabetes, and it is not known what constitutes glycemic happiness to them in the context of diabetes care.

Against this background, an effort was undertaken to understand and define “glycemic happiness” in persons living with type 2 diabetes mellitus (T2DM) and assess the patient-, physician-, caregiver-, nurse-, and diabetes counselor-related factors that influence it. An expert group meeting was convened to discuss the concept of glycemic happiness along with the factors affecting it, based on a thorough literature review.

This document summarizes the background evidence-based discussion, key opinions, and views of experts on the concept of glycemic happiness, and presents the design delineated by experts for a proposed observational survey to understand what constitutes glycemic happiness and the factors influencing it.

## 2. Materials and Methods

An expert group meeting was held with a group of nine expert diabetologists and endocrinologists from India in September 2019. The purpose of the meeting was to define and discuss the topic of glycemic happiness and to delineate the parameters that could be used to evaluate the glycemic happiness of persons living with diabetes. After a comprehensive review of various available validated scales and discussion based on practical clinical experience, the experts developed five sets of 5-point Likert method-based questionnaires that may help assess the “glycemic happiness” of the patient. One questionnaire was developed for persons with T2DM, and the remaining four questionnaires were designed for the physicians, caregivers, nurses, and diabetes counselors/educators, to help understand the factors that affect the glycemic happiness of the patient either directly or indirectly. Finally, an observational glycemic happiness survey was proposed to be conducted based on the questionnaires developed for the assessment of the patient-, physician-, caregiver-, and HCP-related factors, which influence and define the glycemic happiness of persons with T2DM.

### 2.1. Psychological Outcomes in Persons with Diabetes

#### 2.1.1. Challenges for Persons Living with Diabetes

Psychosocial challenges are most common among persons with diabetes, and if left unattended, can seriously impact the person’s social life and well-being. Addressing these concerns through treatment interventions would help overcome psychological barriers, thereby improving self-care and adherence to treatment, which is the ultimate goal of management in diabetes [1]. The psychosocial challenges for persons with diabetes are listed in Table 1.

#### 2.1.2. Significance of Psychological Impact of Diabetes

Being diagnosed with diabetes can be depressing and challenging. This can impact the glycated hemoglobin (HbA_1c_) levels, as these are affected by emotions similarly to any antidiabetic medication. To reflect the experience of a person living with diabetes, it has been compared to a job, where a person must work 24/7, 365 days a year, without any praise or pay [11].

For a more vivid picture of the psychological impact of diabetes, the diagnosis of diabetes has been compared with grief experienced when someone close is lost. Being diagnosed with diabetes prompts the grieving of one’s lost health [11]. Following the diagnosis of diabetes, its management requires lifelong day-to-day adherence to exercise and dietary plans, frequent blood glucose monitoring, and medications. All these strict regimens augment the risk of lowered physical and emotional well-being among persons living with diabetes [12]. Various psychological effects associated with diabetes include depression, diabetes burnout, anxiety, fear of hypoglycemia, fear of needles, eating issues, insulin omission, poor communication between patients and their physicians and caregivers, disturbed family relationships, and other lifestyle changes [11]. The psychological impact of diabetes leads to poor quality of life (QoL), nonadherence to medications, poor self-management, and lack of interest in diabetes management, culminating in long-term complications and poor glycemic control [12]. Evidence indicates that the day-to-day QoL in persons living with diabetes can be greatly improved by decreasing the psychological burden of diabetes [13].

#### 2.1.3. Clinical Evidence on the Impact of Diabetes Burden on QoL in Persons Living with Diabetes

A study explored the association of psychosocial factors, such as coping skills, psychological distress, trauma exposure, and family support, with initial weight and weight loss programs among prediabetic people. The findings of the study demonstrated that the weight loss program for the prevention of diabetes in the prediabetic population is significantly affected by psychological factors [14]. Eilander et al. demonstrated that behavior problems in persons living with diabetes are associated with elevated HbA_1c_ and result from diabetes mismanagement and low self-confidence [15]. Another study has shown that there exists a positive association between depression and a high level of diabetes distress [16].

Self-management of diabetes, an important component of diabetes care, is severely affected by the psychological burden related to diabetes [12]. Self-management includes parameters such as eye care, daily foot care, oral care, use of blood glucose monitor, knowledge of goals in diabetes management and how to reach them, use of medications as per prescription, and monthly oral self-exam [6]. Hofmann et al. showed that the implementation of self-management intervention positively affects both behavioral and psychological outcomes and decreases diabetes distress in persons living with diabetes [17]. All these studies reaffirm the guidelines of the American Diabetes Association (ADA), which mentions that the medical management of diabetes should also include assessment of the patient’s psychological and social situation to improve QoL and diabetes-related outcomes in this population [18]. A cross-sectional study from Malaysia evaluated the prevalence of anxiety, depression, and stress symptoms in people with T2DM. The study reported a high prevalence of anxiety, depression, and stress symptoms in people with T2DM, where nearly one-third of the population was identified as anxious [19]. A single-center study from a multispecialty hospital reported that medication adherence in people with T2DM significantly improved following verbal counseling on medication adherence [20].

The second Diabetes Attitudes, Wishes and Needs (DAWN2^TM^) study evaluated the psychological outcomes in 8596 persons living with diabetes across 17 countries, including India [21]. According to the study, a negative impact on various aspects of daily living was reported worldwide in persons living with diabetes. Based on the results, approximately half of the patients reported a negative impact on emotional well-being (42.5%). Persons living with diabetes also reported that diabetes had a negative impact on their relationship with family, friends, and peers (21%); physical health (62%); leisure activities (38%); work/studies (35%); and financial situation (44%) and that taking medication interfered with their ability to live a normal life (39%). The study found that only 23% of caregivers or family members participated in any of the diabetes education programs or activities. Furthermore, the majority of the HCPs (63%) felt the need for improved availability of resources for psychosocial support and about 59% of HCPs were in favor of receiving more training in addressing the psychosocial needs of persons living with diabetes [13,21]. Therefore, reducing the burden of diabetes by increasing the psychological support and education could essentially improve the day-to–day QoL [21].

#### 2.1.4. Impact of Diabetes Burden on the QoL in Indian Population: Lessons from the DAWN2^TM^ Study

The DAWN2^TM^ study revealed that healthcare practices in India lagged behind other DAWN2^TM^ countries in the context of diabetes care [22]. As compared to other DAWN2^TM^ countries, healthy self-management, including self-monitoring blood glucose, foot care, and adherence, was ranked poorly in India. Moreover, Indian participants of the study reported low participation in educational programs. The study results also revealed that as compared to other countries, a negative impact of diabetes and experiences of social discrimination were more common in India. The level of active patient engagement, as well as empowerment, was found to be the lowest in India. India had the lowest percentage of respondents participating in any educational program or activities. According to the study, the areas that needed special attention in India for better diabetes care were the number of diabetes instructors and nurses available for diabetes education, access to psychologists and psychiatrists, and communication within the healthcare team [22]. India has a successful model of “seven-sister stakeholders” involvement in diabetes care. These stakeholders are (i) persons living with diabetes, (ii) people who matter, such as friends, family, and colleagues, (iii) the community, (iv) paramedical professionals and physicians, (v) religious leaders, i.e., priests and preachers, (vi) policymakers and planners, including the government and private healthcare systems, and (vii) payers or health insurance reimbursors. The results of the DAWN2^TM^ study reiterate the need for introspection and action from all these stakeholders for improving the quality of diabetes care in the country [22].

Several other cross-sectional Indian studies have also reported a high prevalence of diabetes distress, depression, and stress in the Indian population living with diabetes. Dogra et al. reported a 56.8% prevalence of depression in persons living with diabetes [23]. A study from North India reported 18% prevalence of diabetes distress in persons with T2DM, which included 16.1% of emotion-related distress, 1.5% of interpersonal-related distress, 5.6% of regimen-related distress, and 1.2% of physician-related distress [24]. Another study reported that about 41.9% of persons with T2DM had “high diabetes distress,” which, in turn, significantly affected their adherence to antidiabetic medication [25]. A study from South India reported a high prevalence of diabetes distress, which was largely associated with emotional burden related to the duration of diabetes, regimen-related distress, and poor glycemic control [26]. A meta-analysis of 43 studies involving 10,270 patients reported a 38% prevalence of depression in persons with T2DM in India, which was associated with an increased incidence of complications [27]. Another study from South India highlighted a 35% prevalence of high/very high stress among persons living with T2DM [28]. Another study reported a 77.5% prevalence of moderate to high diabetes-related distress, wherein women had significantly higher distress as compared to men with T2DM [29]. These findings highlight the need for early screening for psychological burden in the Indian population living with T2DM [27].

### 2.2. The Emergence of “Glycemic Happiness” Concept

#### 2.2.1. Introducing the Concept of Glycemic Happiness

Despite the chronic nature of the disease, it is possible for people living with diabetes to lead productive, fulfilling lives, without being overwhelmed by diabetes—this can be achieved through the concept of glycemic happiness. Glycemic happiness intends to shift the focus from the amelioration of diabetes to the promotion of overall well-being in diabetes care. Happiness is an integral component of human health, which serves as a means to overcome challenges and achieve good health [8]. Thus, happiness or euthymia is the purpose of healthcare, including that for persons with diabetes [8]. Rather than as an endpoint of achieving good health, happiness should be viewed as a means to achieve it. While other barriers, such as the burden of diabetes and its stressors, impact a person’s well-being, happiness is mainly determined by the person’s attitude rather than any external factor. Thus, the diabetes care team should also focus on motivation and behavior change in addition to biomedical care [6,8].

#### 2.2.2. The Holy Grail of Diabetes Care: Achieving Glycemic Happiness

The four pillars of glycemic happiness are a happy patient, a happy doctor, a happy caregiver/family, and happy communication between them [8]. Both glycemic control and treatment adherence among patients can be improved through psychological interventions, such as motivational therapy, behavioral therapy, coping skills training, problem-solving therapy, and family behavior therapy. Apart from the clinician, nurses and diabetes counselors also have a crucial role in these psychological interventional practices. Strategies focused on reducing disease burden and improving diabetes outcomes can be formulated by clinicians with an increased understanding of the psychological aspects of persons living with diabetes [1]. For diabetes caregivers, both verbal and nonverbal communication styles significantly contribute to the happiness of the person with diabetes. A happy diabetes caregiver generates a sense of optimism in the person living with diabetes, which can be achieved through proactive and friendly communication [8]. The proactive involvement of clinicians, nurses, and diabetes counselors can help achieve glycemic happiness. The expert perspectives on glycemic happiness are summarized in Box 1.

Box 1. **Glycemic Happiness: Expert Perspective.**

- Due to the huge burden of diabetes mellitus in India, the introduction of the concept of glycemic happiness is very essential.
- Definition: Glycemic happiness is “a state of emotional and biomedical well-being in persons with diabetes mellitus”.
- Glycemic happiness can be achieved by targeting factors that account for the well-being of persons with diabetes.

### 2.3. Quantifying Glycemic Happiness: Developing a “Glycemic Happiness Scale”

The detrimental consequences of psychological “unhappiness” are not inevitable. However, an essential prerequisite for addressing such unhappiness is the early detection of the problem [30]. Indeed, routine screening for psychological and psychosocial symptoms is recommended by the ADA guidelines [18]. However, it was estimated that in clinical practice, only 25% of depressed persons living with diabetes are identified. Therefore, the identification of different aspects of psychological unhappiness is challenging in the clinical setting. Multiple questionnaires have been developed to estimate diabetes-specific stressors, such as depression [30].

#### 2.3.1. Available Tools and Scales for Measuring Happiness and Well-Being in Patients

The available happiness scales are not specific to diabetes. In diabetes settings, though various scales are available, none of them measure glycemic happiness. Therefore, there is a need for a tool to evaluate the glycemic happiness of a person living with diabetes. The different scales available for quantifying different aspects of happiness or well-being in the nondiabetic and diabetic settings are described below.

##### In Nondiabetic Setting

- The World Health Organization (WHO)-5 Well-Being Index: World Health Organization-5 (WHO-5) is the most widely used questionnaire that measures the dimensions of psychological general well-being. This is a five-item scale that comprises a short questionnaire (five questions). The questions are based on how the person has felt over the past two weeks, with the scores ranging from 0 (at no time) to 5 (all of the time). The feelings of psychological well-being are gauged through questions on cheerfulness, calmness, proactiveness, etc. [31].

The total score ranges from 0 to 25, where 0 indicates the worst possible and 25 indicates the best possible QoL. To obtain a percentage score ranging from 0 to 100, the raw score is multiplied by 4, where a percentage score of 0 indicates the worst possible QoL, whereas a score of 100 indicates the best possible QoL. This scale has ample validity both as a screening tool and outcome measure for depression and has been successfully applied in a variety of studies [31].

- Happiness Index: The Happiness Index scores the happiness of countries on a scale from 1 to 10, where a score of 0 indicates “not at all happy”, while a score of 10 indicates “extremely happy”. The Happiness Index ranges from 0 to 200 and is defined as the weighted rate of respondents reporting “quite happy” or “very happy” less the weighted rate of respondents reporting “not very happy”. India’s happiness ranking is falling consistently. What makes this worse for India is that the country slipped a further 11 places in the 2018 report as compared to 2017, where it dropped by 4 places. In 2017, India was ranked 122nd on the list, whereas, in 2016, it was placed at 118 [32].
- The Well-Being Questionnaire-12: The 12-item well-being questionnaire was developed based on the balance between positive- and negative-worded items. It consists of three four-item subscales, including positive well-being, energy, and negative well-being. Four items that define negative well-being include crying spells, downhearted and blue, afraid for no reason, and upset or feeling panicky. These four items generate the total negative well-being score ranging between 0 and 12. A higher score is indicative of a greater feeling of negative well-being. The four items that define energy are energetic, fresh and rested, dull, and tired. The scores for the last two items are reversed and then summed together with the other two items to produce a total energy score ranging between 0 and 12, whereby a higher score is indicative of greater energy levels. The four items defining positive well-being are happy with life, live life I want to, tackle daily tasks, and cope with problems. The scoring principle for positive well-being is similar to that of negative well-being scoring. The 12-item well-being questionnaire is a short, valid, and reliable measure of psychological well-being among patients [33].
- Patient Health Questionnaire: The 9-item Patient Health Questionnaire evaluates the degree of depression severity. Each of the nine items in the questionnaire is rated on a scale of 0 (never) to 3 (almost every day), and the total score is 27. For any patient, if the total score is ≥10, it indicates major depressive disorder and the person should be referred to a clinic. Overall, a lower score indicates better psychological and physical health, while a higher score indicates higher severity of depression in the patients. Therefore, this scale identifies both depression and the severity of depression [34].
- The Work and Social Adjustment Scale: The Work and Social Adjustment Scale was developed to measure impairment in functioning, which is attributable to an identified problem. It is a five-item scale, where each item is rated on a scale of 0 to 8. A score of 0 indicates “not at all impaired” and a score of 8 indicates “very severely impaired”. This scale permits the comparison of functioning impairment across studies and for multiple disorders [35].

The five detailed questionnaires for measuring happiness in a nondiabetic context, as described above, are presented in Table 2.

##### In Diabetic Setting

- The Appraisal of Diabetes Scale: The 7-item Appraisal of Diabetes Scale, a self-report questionnaire, evaluates an individual’s appraisal of diabetes [36], i.e., an individual’s thoughts regarding coping with diabetes [37].
- The Diabetes Distress Scale: The 17-item Diabetes Distress Scale is scored based on a 6-point Likert scale. While a score of 6 depicts “a very serious problem”, a score of 1 reflects “no problem” [38,39].
- The GlucoCoper Tool: The 6-item GlucoCoper tool evaluates four positive (acceptance, planning, optimism, and action) and two negatives (blame and resistance) coping mechanisms in people with T2DM. Each of the six items is measured on a 10-point Likert scale. The GlucoCoper tool provides a total score, a negative scale score, and a positive scale score [40].
- Hypoglycemia Attitudes and Behavior Scale: The 14-item Hypoglycemia Attitudes and Behavior Scale evaluates two important aspects of hypoglycemia-related concerns, i.e., avoidance and anxiety, and one positive aspect of confidence [39,41].
- Problem Areas in Diabetes: The 20-item Problem Areas in Diabetes Scale evaluates emotional stress, such as anger, worry, depressed mood, guilt, and fear in persons living with diabetes [42,43].
- Hypoglycemic Confidence Scale: The 9-item Hypoglycemic Confidence Scale evaluates the extent to which persons living with diabetes feel secure, able, and comfortable regarding their ability to stay safe from hypoglycemia-related issues [39,44].
- Diabetes Treatment Satisfaction Questionnaire: The 8-item Diabetes Treatment Satisfaction Questionnaire is a scale that measures satisfaction in treatment regimens in people with T1DM and T2DM [37,45].
- Self-Efficacy for Diabetes Scale: The 8-item Self-Efficacy for Diabetes Scale is widely used to evaluate diabetes-specific self-efficacy. It has three subscales: medical self-efficacy, diabetes-specific self-efficacy, and general self-efficacy [46,47].

The eight detailed questionnaires for measuring different parameters in the context of diabetes as described above are presented in Table 3.

#### 2.3.2. Scales for Assessing Compassion Fatigue, Burnout, and Quality of Communication in Physicians

- Self-assessment Compassion Fatigue Scale: The burnout that manifests itself as emotional, physical, and spiritual exhaustion is defined as compassion fatigue. It is observed among physicians treating diabetes. The common causes contributing to compassion fatigue are lack of time, seeing more patients, and more paperwork. Compassion fatigue can be evaluated using the 9-item self-assessment scale, which determines the risk of compassion fatigue in physicians. The nine questions of this scale are answered with either “yes” or “no”, and when the answer is “yes” for ≥4 questions, it indicates that the physician might be having compassion fatigue [48].
- Professional Quality of Life Scale: The 30-item Professional Quality of Life Scale measures the positive and negative experiences of the current work situation as a helper in the last 30 days. Each of the 30 items is rated on a scale of 1 to 5; while a score of 1 indicates “never”, a score of 5 reflects “very often” [49]. This scale is used to measure the positive and negative effects of helping others experiencing trauma and suffering [50].
- Professional Fulfilment Index: The 16-item Professional Fulfilment Index measures professional fulfillment and burnout, particularly for sensitivity to changes that are attributable to interventions or other factors that affect physician’s well-being. The 16 items of the scale are divided into three parameters, four items for work exhaustion, six items for professional fulfillment, and six items for interpersonal disengagement. Each of the 16 items is rated on a scale of 0 to 4, where score 4 indicates “completely true”, while score 0 indicates “not at all true” [51].
- Quality of Communication Questionnaire: The 19-item Quality of Communication Questionnaire measures how well a physician is taking care of the patient. It helps in improving the communication between the patient and the physician. The questionnaire is meant for the patients to share their feelings regarding how good and comfortable their doctors are in communicating with them. Each of the 19 items is rated on a scale of 0 to 10, where for the first 17 items, a score of 10 indicates “the very best I could imagine” and a score of 0 indicates “the very worst I could imagine”. For the 18th and 19th items, a score of 10 indicates “extremely comfortable” and a score of 0 indicates “not at all comfortable” [52].

The four detailed questionnaires for assessing compassion fatigue, burnout, and quality of communication in physicians, as described above, are presented in Table 4.

The majority of the available scales as listed above measure happiness in physicians. However, none of the available scales evaluate specific parameters related to the physicians that can influence the glycemic happiness of their patients.

#### 2.3.3. Delineating the Glycemic Happiness Evaluating Parameters for Persons Living with T2DM

After a detailed assessment of various scales and tools used globally for the assessment of QoL, the psychological and emotional well-being of patients, communication between patient and doctor, compassion fatigue, and burnout of the physicians, the expert members excerpted the following key messages:

- Correlates of decreased well-being and increased diabetes-related distress negatively impact various aspects of the daily lives of persons with diabetes;
- Factors influencing well-being among persons living with diabetes include poor glycemic control and medication adherence, poor support from healthcare professionals/caregivers, and certain social factors;
- Due to the high burden of diabetes mellitus reported in India, the introduction of the concept of “glycemic happiness” seems essential;
- Glycemic happiness can be achieved by targeting factors that account for the well-being of persons living with diabetes.

Based on the literature review and discussion on various nondiabetic and diabetic scales, the expert panel recommended that the parameters for evaluating glycemic happiness should include elements related to diabetes distress, emotional distress, coping mechanisms, treatment satisfaction, self-efficacy, control over diabetes, and hypoglycemia in persons living with diabetes. Additionally, the panel also reviewed validated scales and questionnaires for assessing compassion, fatigue, burnout of physicians, and quality of communication between physicians and patients.

In addition to factors specific to persons living with diabetes, as derived from the above-validated scales, that may contribute to the “glycemic happiness” of persons with diabetes, other factors related to caregivers, nurses, and diabetes counselors may also influence “glycemic happiness”. Studies indicate a high degree of caregiving burden and depression along with poor psychological health and well-being among Indian diabetes caregivers [53,54].

After a comprehensive review of all the above-validated scales and literature and a discussion based on practical clinical experience, the diabetes expert panel developed five sets of questionnaires with questions that may help correlate with “glycemic happiness”. One questionnaire was for persons with T2DM to help understand what constitutes glycemic happiness to them. The remaining four questionnaires were for the physicians, caregivers, nurses, and diabetes counselors/educators, to help understand the factors that affect the glycemic happiness of the patient either directly or indirectly. Although at present, diabetes is referred to as a chronic health condition rather than a disease [55], in the proposed study protocol and questionnaire, individuals with T2DM have been referred to as “patients” following the common convention, for the ease of understanding of the study participants. Each questionnaire is simple, easy to fill, and as visually appealing as possible with the use of popular emojis and a 5-point Likert scale used to gather responses. The responses to the 5-point Likert scale would range from “strongly agree” to “strongly disagree”. The finalized questionnaires would be translated into various regional languages for easy data collection. The separate questionnaire for each of the five components is depicted in Box 2.

Box 2. **Five-Component Questionnaire of the Glycemic Happiness Scale.**

- Glycemic Happiness Scale for Patient Component
  ▪ How satisfied are you with your understanding of your diabetes?
  ▪ Do you feel that friends or family don’t appreciate how difficult living with diabetes can be?
  ▪ How happy and satisfied are you with your life presently?
  ▪ How flexible have you been finding your treatment to be recently?
  ▪ How convenient have you been finding your treatment to be recently?
  ▪ How confident do you feel that you know what to do when your blood sugar level goes higher or lower than it should be?
  ▪ Do you feel your private and social leisure activities are impaired due to diabetes?
  ▪ Do you feel that diabetes is taking up too much of your mental and physical energy every day?
  ▪ Do you get angry, scared, and/or depressed when you think about living with diabetes?
  ▪ Do you feel overwhelmed by the demands of living with diabetes?
- Glycemic Happiness Scale for Physician Component
  ▪ Do you feel happy and satisfied that you chose to be a diabetes care professional?
  ▪ Do you get satisfaction from being able to help persons with T2DM?
  ▪ Do you feel you can make a difference in the life of persons with T2DM through your work?
  ▪ Do you get physically and emotionally exhausted at work?
  ▪ Do you feel you are losing enthusiasm at work?
  ▪ Do you feel you are in control of dealing with complex problems of T2DM management?
  ▪ Do you feel worn out by your job as a care provider?
  ▪ Do you feel overwhelmed because persons with diabetes’ loads seem endless?
  ▪ Do you feel depressed by the traumatic stress of persons with T2DM that you try to help?
  ▪ Do you feel less empathetic and connected with your colleagues and friends?
- Glycemic Happiness Scale for Caregiver Component
  ▪ Do you get adequate information from your doctor for providing care to your relative who has T2DM?
  ▪ As a caregiver, do you have constructive conversations with the person with type 2 diabetes mellitus, when he or she experiences anxiety?
  ▪ Being a caregiver, do you help the person with T2DM in regular blood glucose monitoring?
  ▪ Do you accompany the person with T2DM during exercise/sports/other physical activity?
  ▪ Do you feel dealing with hypoglycemia is one of the biggest challenges you face when it comes to being a caregiver of a person with T2DM?
  ▪ As a caregiver, do you feel your personal, physical, and mental health is getting affected?
  ▪ Do you feel, you have to give up vacations, hobbies, or other social activities, being a caregiver?
  ▪ Do you feel you can keep your energy levels up while caring for the person with T2DM?
  ▪ Do you get time to relax, while caring for the person with T2DM?
  ▪ How happy and satisfied are you with your life presently?
- Glycemic Happiness Scale for Nurse Component
  ▪ Do you feel happy and satisfied that you chose to be a diabetes care professional?
  ▪ Do you get satisfaction from being able to help persons with T2DM?
  ▪ Do you feel that you can make a difference in the life of persons with T2DM through your work?
  ▪ Do you get physically and emotionally exhausted at work?
  ▪ Do you feel you are losing enthusiasm at work?
  ▪ Do you feel you are in control of dealing with complex problems of T2DM management?
  ▪ Do you feel worn out by your job as a diabetic care provider?
  ▪ Do you feel overwhelmed because your caseload seems endless?
  ▪ Do you feel depressed by the traumatic stress of those you try to help?
  ▪ Do you feel less empathetic and connected with your colleagues and friends?
- Glycemic Happiness Scale for Counselor/Educator Component
  ▪ Do you feel happy and satisfied that you chose to be a diabetes care professional?
  ▪ Do you get satisfaction from being able to help persons with T2DM?
  ▪ Do you feel that you can make a difference in the lives of persons with T2DM through your work?
  ▪ Do you get physically and emotionally exhausted at work?
  ▪ Do you feel you are losing enthusiasm at work?
  ▪ Do you feel you are in control of dealing with complex problems of T2DM management?
  ▪ Do you feel worn out by your job as a diabetic care provider?
  ▪ Do you feel overwhelmed because your caseload seems endless?
  ▪ Do you feel depressed by the traumatic stress of those you try to help?
  ▪ Do you feel less empathetic and connected with your colleagues and friends?

## 3. Proposed Observational Survey for Assessing the Glycemic Happiness of Persons Living with T2DM: Setting the Context

Since there is no standard definition of “glycemic happiness”, the expert panel suggested a cross-sectional observational survey among the persons living with T2DM and the relevant stakeholders using the questionnaires for a better understanding of “glycemic happiness”. The basic layout of the survey is described below.

### 3.1. Aims

To understand what constitutes the glycemic happiness of a person living with T2DM.

### 3.2. Objectives

To understand and define the “glycemic happiness” of persons with T2DM and assess various physician, caregiver, nurse, and diabetes counselor-related factors that influence it.

### 3.3. Study Design and Methodology

A prospective, multicentric, cross-sectional, observational survey will be conducted to understand what constitutes the glycemic happiness of persons living with T2DM. After obtaining the ethical committee approval for the survey, physicians and designated personnel at the site will be trained on the survey procedures. The survey will be conducted for one month. Execution of the survey at the site will be performed as follows:

▪ As per the inclusion criteria, persons with T2DM will be identified by the physician and consent will be obtained from the patient;
▪ After obtaining consent, data will be collected from patient medical records;
▪ Thereafter, designated personnel at the site will seek feedback from patients on the patient component of the glycemic happiness questionnaire;
▪ After receiving the feedback from patients, designated personnel will administer the survey to caregivers accompanying the patients to the clinic/hospital by using the caregiver component of the glycemic happiness questionnaire;
▪ Each site will enroll five patients and five caregivers accompanying the patient;
▪ The site team, i.e., physician, nurse, and diabetes counselor/educator, will self-administer the survey and enter the data in an electronic case report form.

Demographic details (age and gender) will be collected from all the participants by the designated site personnel. In addition, in the case of persons with T2DM, details on height, weight, vital signs, duration of diabetes, and current or last available glycemic indices will be collected, as available. All participants will be asked to rate the parameters in their respective glycemic happiness questionnaires based on their degree of agreement with each parameter on a Likert scale of 1 to 5 (where 1 = Strongly disagree; 2 = Disagree; 3 = Neutral (Neither Agree nor Disagree); 4 = Agree and 5 = Strongly agree). The Investigator/co-Investigator/study coordinator will record the data from the source documents onto the electronic case report form. Data will be presented as mean percentage rating for each “agreement” dimension for each of the parameters derived from the five questionnaires.

### 3.4. Selection Criteria

The inclusion criteria will be (i) persons aged ≥18 years with T2DM, or physicians (endocrinologists/diabetologists/consulting physicians/general physicians), caregivers (family), diabetes nurses or diabetes counselors/educators dealing with persons with T2DM aged ≥18 years; (ii) participants willing to sign the informed consent form and participate in the survey. Participants not willing to sign the informed consent form and participate in the survey will be excluded from the study.

### 3.5. Study Endpoints

▪ Parameters from the patient questionnaire with the highest mean percentage rating, which define “glycemic happiness” in persons with T2DM.
▪ Parameters from the physician questionnaire with the highest mean percentage rating, which positively influence the glycemic happiness of persons living with T2DM.
▪ Parameters from the caregiver questionnaire with the highest mean percentage rating, which positively influence the glycemic happiness of persons living with T2DM.
▪ Parameters from the nurse questionnaire with the highest mean percentage rating, which positively influence the glycemic happiness of persons living with T2DM.
▪ Parameters from the diabetes educator/counselor questionnaire with the highest mean percentage rating, which positively influence the glycemic happiness of persons living with T2DM.
▪ Defining what glycemic happiness is to the patients and understanding the various factors influencing it.

## 4. Conclusions

Apart from long-term complications associated with diabetes, the psychological impact of diabetes also significantly contributes to poor outcomes in persons living with diabetes. The basic problem statement of persons living with diabetes is that they seem to be unhappy in the context of diabetes management. Therefore, it is essential to introduce the concept of glycemic happiness and understand the parameters that determine/influence such happiness. In this context, the expert panel proposed a cross-sectional observational study using a set of questionnaires. The objective of this cross-sectional observational survey is to understand and define the “glycemic happiness” of persons living with T2DM and assess the physician-, caregiver-, nurse-, and diabetes counselor-related factors that influence it. Given the huge burden of diabetes in India, this article is likely to serve as a reminder for the physicians, highlighting the need to address the overall well-being of persons living with T2DM by addressing both metabolic and psychosocial aspects of diabetes management.

## Figures and Tables

**Table 1:** Psychosocial challenges for persons with diabetes [1,9,10].

Lifestyle-related	Behavior modification
	Nutrition Physical activity Abstinence from substance abuse (often related to pressure)
Therapy-related	Monitoring Medications Insulin Regular healthcare visits Economic cost
Complication-related	Risk of complications Increased cost of therapy Multidisciplinary care
Psychosocial	Eating disorders/body image issues

**Table 2:** Available tools and scales for measuring happiness in a nondiabetic setting.

WHO-5 Well-Being Index [31]	Happiness Index [32]	Well-Being Questionnaire-12 [33]	Patient Health Questionnaire [34]	Work and Social Adjustment Scale [35]
Daily life has been filled with things that interest me? Woke up feeling fresh and rested? Felt active and vigorous? Felt calm and relaxed? Felt cheerful and in good spirits?	GDP per capita Social support Healthy life expectancy Freedom to make life choices Generosity Perceptions Unexplained happiness	Neg 1: Crying spells Neg 2: Downhearted and blue Neg 3: Afraid for no reason Neg 4: Upset or feel panicky Energy 1: Energetic Energy 2 (Reversed): Dull Energy 3 (Reversed): Tired Energy 4: Fresh and rested Pos 1: Happy with life Pos 2: Live life I want to Pos 3: Tackle daily tasks Pos 4: Cope with problems	Little interest or pleasure in doing things Feeling down, depressed, hopeless Trouble sleeping or sleeping too much Feeling tired or having little energy Poor appetite or overeating Feeling bad about self, or a failure, or have let self or family down Trouble concentrating, such as reading the newspaper or watching TV Moving or speaking more slowly, or being restless, moving more than usual Thoughts of self-harm	Because of my (problem) my ability to work is impaired Because of my (problem) my home management (cleaning, tidying, shopping, cooking, looking after home or children, paying bills) is impaired Because of my (problem) my social leisure activities (with other people, e.g., parties, bars, clubs, outings, visits, dating, home entertaining) are impaired Because of my (problem), my private leisure activities (done alone, such as reading, gardening, collecting, sewing, walking alone) are impaired Because of my (problem), my ability to form and maintain close relationships with others, including those I live with, is impaired.

WHO: World Health Organization; GDP: gross domestic product; Neg: negative; Pos: positive; TV: television.

**Table 3:** Available tools and scales for measuring different parameters in a diabetic setting.

Appraisal of Diabetes Scale [36]	Diabetes Distress Scale [38]	The GlucoCoper Tool [40]
How upsetting is having diabetes for you? How much control over your diabetes do you have? How much uncertainty do you currently experience in your life as a result of being a person with diabetes? How likely is your diabetes to worsen over the next several years? Do you believe that achieving good diabetic control is due to your efforts as compared to factors that are beyond your control? How effective are you in coping with your diabetes? To what degree does your diabetes get in the way of your developing life goals?	Feeling that diabetes is taking up too much of my mental and physical energy every day. Feeling that my doctor doesn’t know enough about diabetes and diabetes care. Feeling angry, scared, and/or depressed when I think about living with diabetes. Feeling that my doctor doesn’t give me clear enough directions on how to manage my diabetes. Feeling that I am not testing my blood sugars frequently enough. Feeling that I am often failing with my diabetes regimen (Energy 4: Fresh and rested). Feeling that friends or family are not supportive enough of my self-care efforts (e.g., planning activities that conflict with my schedule, encouraging me to eat the “wrong” foods). Feeling that diabetes controls my life. Feeling that my doctor doesn’t take my concerns seriously enough. Not feeling confident in my day-to-day ability to manage diabetes. Feeling that I will end up with serious long-term complications, no matter what I do. Feeling that I am not sticking closely enough to a good meal plan. Feeling that friends or family don’t appreciate how difficult living with diabetes can be. Feeling overwhelmed by the demands of living with diabetes. Feeling that I don’t have a doctor who I can see regularly about my diabetes. Not feeling motivated to keep up my diabetes self-management. Feeling that friends or family don’t give me the emotional support that I would like.	Negativity: How often do you get stuck in extremely negative or persistently negative thoughts? Blame: How often do you blame yourself or others for diabetes? Acceptance: How well do you accept diabetes as a part of your life? Optimism: How often do you have pleasant or positive thoughts? Planning: How well do you plan strategies to manage diabetes? Action: How often do you take positive actions to manage diabetes or to manage life?
**Hypoglycemia Attitudes and Behavior Scale** [39]	**Problem Areas in Diabetes** [43]	**Hypoglycemic Confidence Scale** [39]
To avoid serious problems with low blood sugar, I tend to keep my blood sugars higher than I probably should. I am terrified that I might pass out in public due to a low blood sugar episode. Without even bothering to test, I take quick action to raise my blood sugars at the first hint of any “funny” feelings. I am confident that I can stay safe from serious problems with low blood sugar while driving. If I don’t have plenty of emergency supplies to raise my glucose with me, I won’t leave my house. I am confident that I can avoid serious problems due to low blood sugar when I’m alone. I spend so much time worrying about the possibility of a low blood sugar episode that it makes it hard for me to ever feel happy. I am confident that I can catch and respond to low blood sugar before my blood sugars get too low. I am terrified that I might injure myself or someone else because of a low blood sugar episode. To avoid serious problems due to low blood sugar, I eat or drink a lot more often than I really need to. I am confident that I can stay safe from serious problems with low blood sugar while exercising. To avoid serious problems due to low blood sugar, I stay close to home more than I would really like to. If I think my blood sugar is too low, I’ll start eating and I won’t stop until I feel better. I am confident that I can stay safe from serious problems with low blood sugar while out in public.	Worrying about the future and the possibility of serious complications. Feeling guilty or anxious when you get off track with your diabetes management. Feeling scared when you think about living with diabetes. Feeling discouraged with your diabetes regimen. Worrying about low blood sugar reactions. Feeling constantly burned out by the constant effort to manage diabetes. Not knowing if the mood or feelings you are experiencing are related to your blood glucose. Coping with complications of diabetes. Feeling that diabetes is taking up too much mental and physical energy. Feeling constantly concerned about food. Feeling depressed when you think about living with diabetes. Feeling angry when you think about living with diabetes. Feeling overwhelmed by your diabetes regimen. Feeling alone with diabetes. Feelings of deprivation regarding food and meals. Not having clear and concrete goals for your diabetes care. Uncomfortable interactions around diabetes with family/friends. Not accepting diabetes. Feeling that friends/family are not supportive of diabetes management efforts. Feeling unsatisfied with your diabetes physician.	How confident are you that you can stay safe from serious problems with hypoglycemia: When you are exercising? When you are sleeping? When you are driving? When you are in social situations? When you are alone? In general, how confident are you that you can: 6. Avoid serious problems due to hypoglycemia? 7. Catch and respond to hypoglycemia before your blood sugars get too low? 8. Continue to do things you really want to do in your life, despite the risks of hypoglycemia? 9. If you have a spouse/partner, what is your best guess about how confident your spouse or partner feels about your ability to avoid serious problems due to hypoglycemia?
**Diabetes Treatment Satisfaction Questionnaire** [45]	**Self-Efficacy for Diabetes Scale** [47]	
How satisfied are you with your current treatment? How often have you felt that your blood sugars have been unacceptably high recently? How often have you felt that your blood sugars have been unacceptably low recently? How convenient have you been finding your treatment to be recently? How flexible have you been finding your treatment to be recently? How satisfied are you with your understanding of your diabetes? Would you recommend this form of treatment to someone else with your kind of diabetes? How satisfied would you be to continue with your present form of treatment?	How confident do you feel that you can eat your meals every 4–5 h every day, including breakfast every day? How confident do you feel that you can follow your diet when you have to prepare or share food with other people who do not have diabetes? How confident do you feel that you can choose the appropriate foods to eat when you are hungry (e.g., snacks)? How confident do you feel that you can exercise 15–30 min, 4–5 times a week? How confident do you feel that you can do something to prevent your blood sugar level from dropping when you exercise? How confident do you feel that you know what to do when your blood sugar level goes higher or lower than it should be? How confident do you feel that you can judge when the changes in your illness mean you should visit the doctor? How confident do you feel that you can control your diabetes so that it does not interfere with the things you want to do?	

**Table 4:** Scales for assessing compassion fatigue, burnout, and quality of communication in physicians.

Self-Assessment Compassion Fatigue Scale [48]	Professional Quality of Life Scale [50]
Personal concerns commonly intrude on my professional role. I find even small changes enormously draining. Association with trauma affects me very deeply. I have lost my sense of hopefulness. My colleagues seem to lack understanding. I can’t seem to recover quickly after association with trauma. My patients’ stress affects me deeply. I feel vulnerable all the time. I feel overwhelmed by unfinished personal business.	I am happy. I feel connected to others. I find it difficult to separate my personal life from my life as a helper. I feel trapped by my job as a helper. I feel depressed because of the traumatic experiences of the people I help. I am pleased with how I am able to keep up with helping techniques and protocols. I feel worn out because of my work as a helper. I believe I can make a difference through my work. As a result of my helping, I have intrusive, frightening thoughts. I can’t recall important parts of my work with trauma victims. I am preoccupied with more than one person I help. I jump or am startled by unexpected sounds. I am not as productive at work because I am losing sleep over the traumatic experiences of a person I help. Because of my helping, I have felt “on edge” about various things. I feel as though I am experiencing the trauma of someone I have helped. I am the person I always wanted to be. I have happy thoughts and feelings about those I help and how I could help them. I avoid certain activities or situations because they remind me of the frightening experiences of the people I help. I feel bogged down by the system. I am a very caring person. I get satisfaction from being able to help people. I feel invigorated after working with those I help. I think that I might have been affected by the traumatic stress of those I help. I like my work as a helper. I have beliefs that sustain me. My work makes me feel satisfied. I feel overwhelmed because my case (work) load seems endless. I am proud of what I can do to help. I have thoughts that I am a “success” as a helper. I am happy that I chose to do this work.
**Professional Fulfillment Index [51]**	**Quality of Communication Questionnaire [52]**
Professional fulfillment (six items) I feel happy at work. I feel worthwhile at work. My work is satisfying to me. I feel in control when dealing with difficult problems at work. My work is meaningful to me. I’m contributing professionally (e.g., patient care, teaching, research, and leadership) in the ways I value most. Interpersonal disengagement (six items) 7. Less empathetic with my patients 8. Less empathetic with my colleagues 9. Less sensitive to others’ feelings/emotions 10. Less interested in talking with my patients 11. Less connected with my patients 12. Less connected with my colleagues Work exhaustion (four items) 13. A sense of dread when I think about work that I have to do 14. Lacking enthusiasm at work 15. Physically exhausted at work 16. Emotionally exhausted at work	Using words that you can understand Looking you in the eye Including your loved ones in decisions about your illness and treatment Answering all your questions about your illness and treatment Listening to what you have to say Caring about you as a person Giving you his/her full attention Talking with you about your feelings concerning the possibility that you might get sicker Talking to you about the details concerning the possibility that you might get sicker Talking to you about how long you might have to live Talking to you about what dying might be like Talking with your loved ones about what your dying might be like Involving you in the decisions about the treatments that you want if you get too sick to speak for yourself Asking about the things in life that are important to you Respecting the things in your life that are important to you Asking about your spiritual or religious beliefs Respecting your spiritual or religious beliefs How comfortable do you feel your doctor is talking about dying? Overall, how would you rate this doctor’s communication with you?

## Data Availability

This article is based on previously conducted studies and does not contain any studies with human participants or animals performed by any of the authors.
